# Phylogenetic analysis of apicomplexan parasites infecting commercially valuable species from the North-East Atlantic reveals high levels of diversity and insights into the evolution of the group

**DOI:** 10.1186/s13071-018-2645-7

**Published:** 2018-01-25

**Authors:** Raquel Xavier, Ricardo Severino, Marcos Pérez-Losada, Camino Gestal, Rita Freitas, D. James Harris, Ana Veríssimo, Daniela Rosado, Joanne Cable

**Affiliations:** 1 CIBIO, Universidade do Porto, Campus Agrário de Vairão, Rua Padre Armando Quintas, 4486-661 Vairão, Portugal; 2 Piscicultura Vale da Lama, Sapal do Vale da Lama, Odiáxere, Lagos, 8600-258 Nigeria; 3 Instituto de Investigaciones Marinas, Consejo Superior de Investigaciones Científicas (IIM-CSIC), Eduardo Cabello, 6, 36208 Vigo, Spain; 4 Virginia Institute of Marine Science, College of William and Mary, Route 1208, Greate Road, 23062 Gloucester Point, Virginia, USA; 5 School of Biosciences, Cardiff University, Cardiff, CF10 3AX UK; 6 Department of Invertebrate Zoology, US National Museum of Natural History, Smithsonian Institution, Washington, DC 20013 USA

**Keywords:** 18S rRNA, Apicomplexa, *Goussia*, *Eimeria*, Aquatic pathogens

## Abstract

**Background:**

The Apicomplexa from aquatic environments are understudied relative to their terrestrial counterparts, and the seminal work assessing the phylogenetic relations of fish-infecting lineages is mostly based on freshwater hosts. The taxonomic uncertainty of some apicomplexan groups, such as the coccidia, is high and many genera were recently shown to be paraphyletic, questioning the value of strict morphological and ecological traits for parasite classification. Here, we surveyed the genetic diversity of the Apicomplexa in several commercially valuable vertebrates from the North-East Atlantic, including farmed fish.

**Results:**

Most of the sequences retrieved were closely related to common fish coccidia of *Eimeria*, *Goussia* and *Calyptospora.* However, some lineages from the shark *Scyliorhinus canicula* were placed as sister taxa to the *Isospora*, *Caryospora* and *Schellakia* group. Additionally, others from *Pagrus caeruleostictus* and *Solea senegalensis* belonged to an unknown apicomplexan group previously found in the Caribbean Sea, where it was sequenced from the water column, corals, and fish. Four distinct parasite lineages were found infecting farmed *Dicentrarchus labrax* or *Sparus aurata*. One of the lineages from farmed *D. labrax* was also found infecting wild counterparts, and another was also recovered from farmed *S. aurata* and farm-associated *Diplodus sargus*.

**Conclusions:**

Our results show that marine fish apicomplexans are diverse, and we highlight the need for a more extensive assessment of parasite diversity in this phylum. Additionally, parasites recovered from *S. canicula* were recovered as basal to their piscine counterparts reflecting hosts phylogeny.

**Electronic supplementary material:**

The online version of this article (10.1186/s13071-018-2645-7) contains supplementary material, which is available to authorized users.

## Background

Parasites from the phylum Apicomplexa are considered some of the world’s most successful parasites, present in most species and responsible for many important diseases [1]. These include *Plasmodium* species, the causative agents of malaria, and *Toxoplasma gondii*, which infects most warm-blooded vertebrates and has a high prevalence in humans [2]. Knowledge across the phylum, however, is extremely patchy. Although the Apicomplexa is the largest group of unicellular obligate parasites with more than 6000 described species, these are estimated to correspond to only 0.1% of the total diversity [1]. Nonetheless, despite their ubiquity and medical/veterinarian importance, the evolutionary history of this group is only now starting to be untangled [3]. Phylogenomic analysis based on 85 proteins indicated that the Apicomplexa evolved from a free-living ancestor since their sister group was identified as free-living chromerids (photosynthetic) and colpodellids (predatory) [4]. Moreover, the Apicomplexa was split into four groups including the most basal cryptosporidean (e.g. *Cryptosporidium*), followed by the coccidians (e.g. *Eimeria*, *Goussia*, *Calyptospora* and *Toxoplasma*), and the sister groups of the haemosporidians (e.g. *Plasmodium*) and piroplasmids (*Babesia* and *Theileria*) (see [4]).

Phylogenetic inference based on the 18S ribosomal RNA gene (18S rRNA) suggests that the Apicomplexa infecting aquatic organisms form a basal group within the family Eimeriidae [5]. This was confirmed by three pivotal studies which focused on the genetic diversity of fish-infecting coccidia from the genera *Eimeria*, *Goussia* and *Calyptospora* (see [6–8]), that led to the hypothesis that terrestrial counterparts evolved from piscine coccidians. Although some of the hosts included in these previous studies can inhabit brackish waters and some are catadromous, almost all sequenced specimens of *Eimeria* (see [6]) and *Goussia* (see [7, 8]) were found infecting hosts collected in freshwater habitats in Hungary. There are only a few exceptions: *Fundulus grandis* collected from the Gulf coast of the Mississippi [7]), *Taurulus bubalis* from an unknown locality [6], and *Selar crumenophthalmus*, *Lutjanus kasmira* and *Mulloidichthys* sp. from Hawaii [8, 9]. For *Calyptospora*, from the three species for which genetic data are available, only one (*C. funduli*) was sequenced from a coastal fish (*F. grandis*; see [8]). The genetic diversity of coccidia infecting fish has been largely neglected; for marine fish there is just a description of an *Eimeria* sp. from farmed Asian sea bass [10], later identified as *Goussia kuehae* [11], the genetic characterization of *G. clupearum* from Atlantic herring [12], and a description of Apicomplexa spp. from Caribbean reef fish (*Stegastes* spp. and *Ophioblennius macclurei*) [13].

The taxonomic uncertainty associated with some *Eimeria* and *Goussia* species and the paraphyly of both genera has been well studied (e.g. [6, 7]). Although most species described under *Eimeria* are phylogenetically related (as suggested by 18S rRNA data), species of *Caryospora* (infecting birds and reptiles), *Schellakia* (infecting reptiles) and *Lankesterella* (infecting amphibians) are clustered in the same *Eimeria* clade [6]. The genus *Goussia* also seems to be paraphyletic and is divided into at least three major groups (*sensu* Rosenthal et al. [7]): the “dispersed” type (e.g. *G. carpelli*), found in the gut; the “nodular” type (e.g. *G. balatonica*), which develop in nodules within the intestine; and the “epicellular” type, present in enterocytes of the gut and kidneys (e.g. *Goussia pannonica*). Although *Calyptospora* is estimated to be monophyletic, the genetic data available are still limited (only three sequences, see [8]).

Despite posing a serious threat to aquaculture (e.g. [14, 15]), there is little information regarding the diversity of coccidian lineages infecting farmed species or farm-associated fish (i.e. those species that enter farms to take advantage of food availability; see [10]). This is surprising given that farming practices can induce increased parasite virulence, for example (i) by selecting for short host lifespan which in turn prompts for accelerated parasite life history (as in the case of Marek’s disease in farmed chicken [16]); (ii) high host densities which facilitate transmission (e.g. [17]); (iii) through vaccination and treatment protocols which reduce the disease symptoms rather than eliminate pathogens [18], thereby relaxing competition between parasites and favouring outbreaks of more virulent opportunistic strains [17]; or (iv) through inbreeding depression of stocks which favours the emergence of specialised pathogen strains (see review by [18]).

In this study, we surveyed the genetic diversity and phylogenetic affinities of coccidians infecting wild and farmed fish from the North-East Atlantic. We aim (i) to build upon the limited knowledge regarding the genetic diversity of coccidian parasites infecting marine vertebrates, and (ii) to determine if farmed fish and farm-associated fish species host genetically divergent parasite lineages.

## Methods

### Sample collection, DNA extraction and PCR amplification

A total of 148 teleosts and elasmobranchs were bought opportunistically from local fish markets and supermarkets in northern Portugal (Labruge and Vila do Conde): wild (*n* = 76), farmed (*n* = 38) and farm-associated (*n* = 34) (see Table 1 for details). Fish were transported to the laboratory and kept frozen at -20 °C until processed. Fish were thawed and dissected, and tissues from internal organs were preserved in 96% ethanol. For wild teleosts, we screened the following internal organs: intestine, stomach, ovaries/testicles, gall bladder, stomach, liver, kidney, spleen, heart and gills, and for elasmobranchs the liver, stomach, kidney, anal gland and testicles/ovaries. From farmed and farm-associated specimens, only the intestines were analysed. Genomic DNA was extracted from each tissue using Jetquick Tissue DNA Spin Kit (Genomed, Lohne, Germany) following the manufacturer’s instructions. The primers published by [19] and designed to amplify *Hepatozoon* were used in a polymerase chain reaction (PCR) to amplify a 600 bp portion of the 18S rRNA gene (HepF300: 5′-GTI TCT GAC CTA TCA GCT TIC GAC G-3′; Hep900 5′-C AAA TCT AAG AAT TIC ACC TCT GAC-3′), following these steps: 3 min at 94 °C, then 30 cycles of 94 °C for 30 s, 60 °C for 30 s and 72 °C for 30 s; and a final extension at 72 °C for 7 min. The 18S rRNA gene was chosen as is the preferred gene for species identification and phylogenetics of the Apicomplexa as sufficient data are available for comparative purposes (e.g. [7]). PCR master mix reactions were performed using Platinum *Taq* and a final MgCl_2_ concentration of 1.5 mM. PCR amplicons were sent for sequencing in both directions by a commercial company Genewiz (Takeley, UK). All new sequences were submitted to the GenBank database (accession nos. MF468290–MF468328).

**Table 1:** List of screened hosts, their origin and the number of infected hosts

Host species	Origin	No. of hosts infected/ No. of hosts analysed
*Trachurus trachurus*	Wild	2/2
*Thunnus* sp.	Wild	1/1
*Scomber japonicus*	Wild	1/2
*Solea senegalensis*	Wild	2/3
*Pagrus caeruleostictus*	Wild	2/7
*Trispterus luscus*	Wild	4/4
*Sparus aurata*	Wild	0/5
*Dicentrarchus labrax*	Wild	1/5
*Chelon labrosus*	Wild	0/2
*Lepidopus caudatus*	Wild	0/1
*Oncorhynchus mykiss*	Wild	0/4
*Platichthys flesus*	Wild	0/1
*Sardina pilchardus*	Wild	0/3
*Dicentrarchus punctatus*	Wild	0/1
*Scyliorhinus canicula*	Wild	6/33
*Raja undulata*	Wild	0/1
*Raja clavata*	Wild	0/1
*Dicentrarchus labrax*	Farmed	4/22
*Sparus aurata*	Farmed	1/16
*Diplodus sargus*	Farm-associated	2/5
*Diplodus annularis*	Farm-associated	0/1
*Diplodus vulgaris*	Farm-associated	0/2
*Chelon labrosus*	Farm-associated	0/8
*Halobatrachus didactylus*	Farm-associated	0/1
*Sardina pilchardus*	Farm-associated	0/1
*Liza ramada*	Farm-associated	0/6
*Mugil cephalus*	Farm-associated	0/1
*Dicentrarchus punctatus*	Farm-associated	0/9

### Sequence alignment and phylogenetic analysis

Sequences were checked manually using the software Geneious v4.8.5 [20] and compared against the GenBank database to confirm whether they belonged within the Apicomplexa. Following the criteria of Rosenthal et al. [7] only those sequences with clean chromatograms were included for analysis. All alignments were performed using MAFFT v7 [21], and the software JModeltest v2.2.1 [22] was used to select the appropriate model of evolution (AIC criteria). Phylogenetic reconstruction analyses based on Maximum Likelihood (ML) and Bayesian Inference (BI) were conducted using Garli v2.1 [23] and MrBayes v3.2.6 [24], respectively.

First, a phylogenetic analysis was conducted including 40 newly generated sequences, plus 18S rRNA gene sequences of coccidians retrieved using the BLAST algorithm against the GenBank database (e.g. apicomplexans infecting corals) and sequences of piscine apicomplexans (included for example in the analyses of [6–8]). Separate phylogenetic analyses were conducted for subsets of related sequences identified in the preliminary analysis to improve alignment quality and outgroup choice. Two parallel runs were conducted for all analyses in MrBayes. In the first preliminary dataset runs were set to 30 million generations, and in the other subsequent analyses to 10 million. The software Tracer v1.6 [25] was used to check for adequate mixing and convergence of each run. Trees from a stationary distribution (25% ‘burn-in’) were used to construct a majority rule consensus tree. For the ML analyses, 1000 bootstraps were used to evaluate branching support. Uncorrected p-distances were calculated for each group using the software MEGA6 [26] to evaluate interspecific divergence and draw taxonomic conclusions.

## Results

Clean nucleotide sequences were derived from apicomplexans collected from multiple organs of nine hosts, including the elasmobranch *Scyliorhinus canicula* (summarised in Table 2). The sequence retrieved from the intestine of *Thunnus* sp. was only 277 bp and was not included in the phylogenetic analysis; however, it was identical to sequences of Eimeriidae gen. sp. retrieved from *Trisopterus luscus* (see Table 2). The remaining sequence was too noisy, probably due to length variants of ribosomal copies, multiple infections or contamination with other organisms (e.g. fungi). Similarly, amplification but noisy sequences were obtained for samples collected from the intestine of *S. senegalensis*, the kidney of *P. caeruleostictus*, liver of *S. japonicus*, gall bladder and stomach of *T. thunnus*, gills of *T. luscus*, the gills, liver, kidney, spleen and stomach of *D. labrax*, kidney of *S. aurata*, and the spleen of *O. mykiss*. The final alignment (including all sequences generated plus relevant sequences deposited on GenBank) was trimmed to 628 bp (including gaps), and was analysed using the GTR + I + G model of evolution. This analysis indicated that the sequences obtained in this study could be divided into five major groups (Additional file 1: Figure S1 and Additional file 2: Figure S2): *Goussia* spp., *Eimeria* spp., *Calyptospora* spp., a fourth group including species of *Caryospora*, *Isospora*, *Schellakia* and *Eimeria*, so far only known to infect amphibians, reptiles, birds and mammals; and, finally, a group of yet unknown apicomplexans. Due to the high diversity and divergence of taxa included in this analysis which limited phylogenetic resolution, additional phylogenetic analyses were performed separately for subsets of sequences representative of these groups, including only unique haplotypes. Sequences for *Eimeria* spp. and *Calyptospora* spp. are usually recovered in phylogenetic analyses as closely related (e.g. [8]), and for this reason, were merged in the same analysis.

**Table 2:** List of infected hosts, infected tissues, phylogenetic affinities of the parasites found and GenBank ID. All hosts have wild origin unless indicated otherwise

Host species	Infected organ	Parasite	GenBank ID
*Diplodus sargus* ^a^	Intestine	*Goussia* sp. (Fig. 1, Additional file 1: Figure S1)	MF468321
*Sparus aurata* ^b^	Intestine	*Goussia* sp. (Fig. 1, Additional file 1: Figure S1)	MF468322
*Scomber japonicus*	Heart, kidney	*Goussia* sp. (Fig. 1, Additional file 1: Figure S1)	MF468319, MF468320
*Dicentrarchus labrax* ^b^	Intestine	*Goussia* sp. (Fig. 1, Additional file 1: Figure S1)	MF468318
	Intestine	*Eimeria* cf. *variabilis* (Fig. 2, Additional file 2: Figure S2); *Eimeria* sp. (Fig. 2, Additional file 2: Figure S2)	MF468291, MF468292
*Dicentrarchus labrax*	Intestine	*Eimeria* sp. (Fig. 2, Additional file 2: Figure S2)	MF468293
*Trisopterus luscus*	Intestine	*Eimeria* sp. (Fig. 2, Additional file 2: Figure S2)	MF468290
	Stomach, gall bladder, liver, heart, spleen, intestine	Eimeriidae gen. sp. (Fig. 2, Additional file 2: Figure S2)	MF468299–MF468308
*Trachurus trachurus*	Intestine, liver, spleen, stomach	Eimeriidae gen. sp. (Fig. 2, Additional file 2: Figure S2)	MF468309–MF468314
*Scyliorhinus canicula*	Liver	Eimeriidae gen. sp. (Fig. 2, Additional file 2: Figure S2)	MF468294
	Liver	*Eimeria* sp. (Fig. 2, Additional file 2: Figure S2)	MF468298
	Liver, anal gland, stomach	Coccidia sp. (Fig. 3, Additional file 2: Figure S2)	MF468295–MF468297
*Pagrus caeruleostictus*	Gall bladder, liver, intestine	*Calyptospora* sp. (Fig. 2, Additional file 2: Figure S2)	MF468315–MF468317
	Heart, kidney	Apicomplexa fam. gen. sp*.* (Fig. 4, Additional file 1: Figure S1)	MF468323, MF468324
*Thunnus* sp.	Intestine	Eimeriidae gen. sp.	MG724744^c^
*Solea senegalensis*	Testes, liver, gills	Apicomplexa fam. gen. sp*.* (Fig. 4, Additional file 1: Figure S1)	MF468325–MF468328

^a^Farm-associated hosts

^b^Farmed hosts

^c^The sequence obtained from the parasite infecting *Thunnus* sp. was not used in the phylogenetic analysis due to its short length

**Fig. 1:**
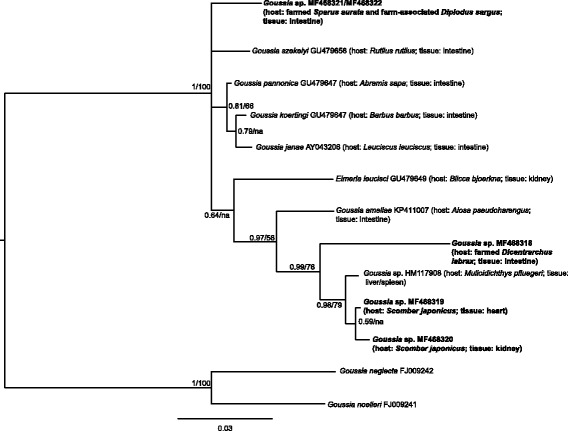
Bayesian inference analysis based on sequences for epicellular *Goussia* *sensu* Rosenthal et al. [7]. Node support values correspond to posterior probabilities and consensus support (%) obtained from Bayesian inference and Maximum Likelihood analyses, respectively. The newly generated sequences are indicated in bold. Only unique haplotypes were included in the analysis

**Fig. 2:**
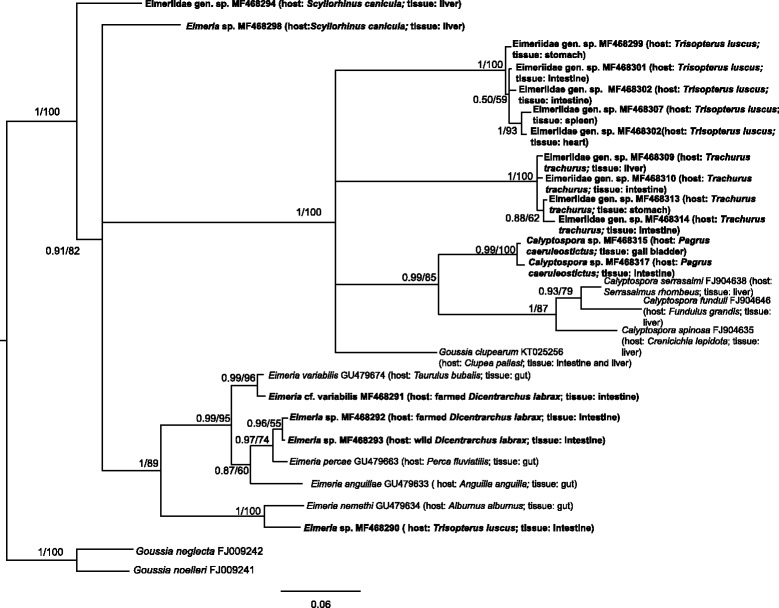
Bayesian inference based on sequences for *Eimeria* and *Calyptospora*. Node support values correspond to posterior probabilities and consensus support (%) obtained from Bayesian inference and Maximum Likelihood analyses, respectively. The newly generated sequences are indicated in bold. Only unique haplotypes were included in the analysis. Host species and infected organs or tissues were detailed for aquatic hosts

**Fig. 3:**
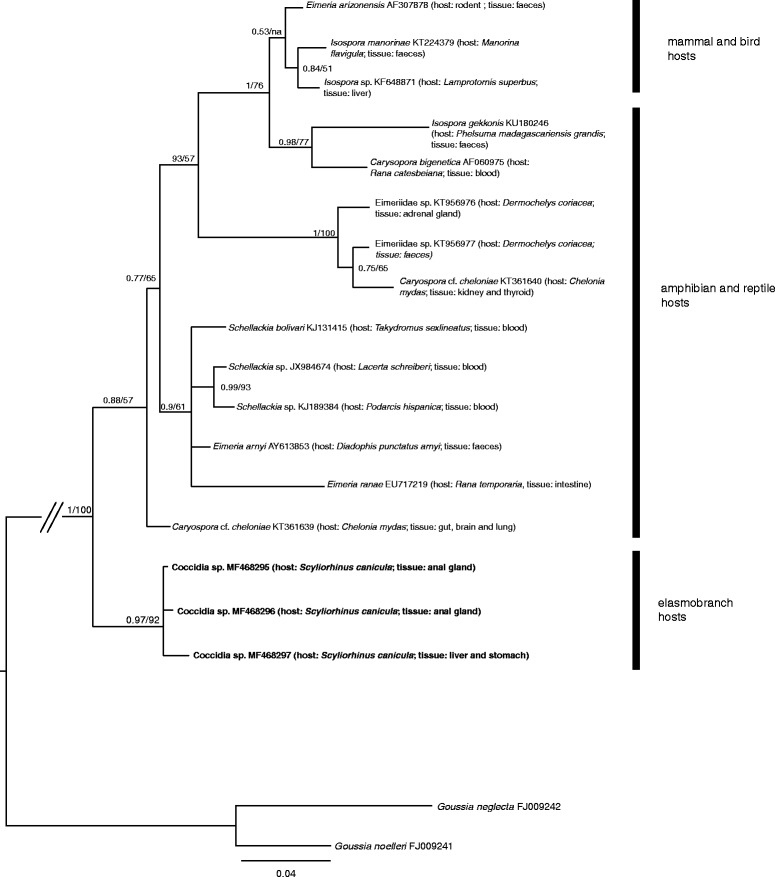
Bayesian inference of phylogenetic analysis of sequences of the *Caryospora* group. Node support values correspond to posterior probabilities and consensus support (%) obtained from Bayesian Inference and Maximum Likelihood analyses, respectively. Major lineages and respective hosts are highlighted. The newly generated sequences are indicated in bold. Unique haplotypes were included in the analysis. Host species and infected organs or tissues were detailed for aquatic hosts

**Fig. 4:**
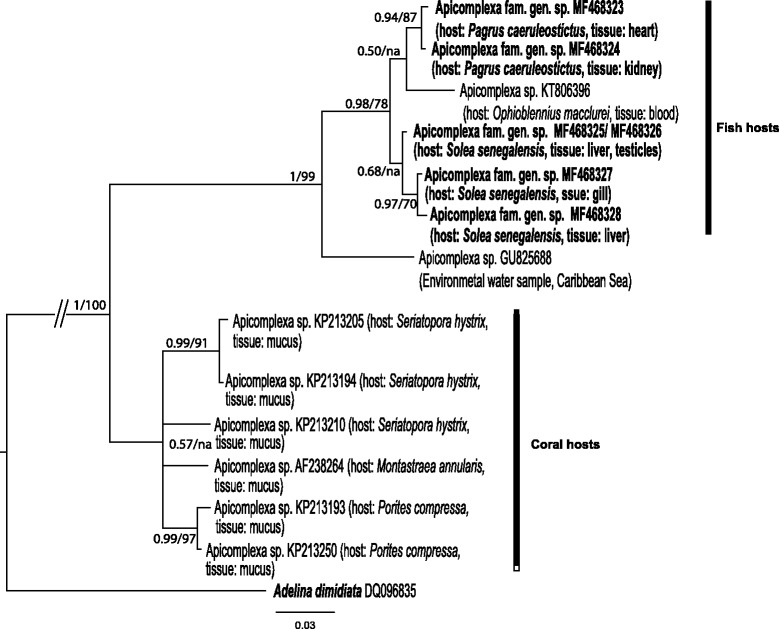
Bayesian inference analysis based on sequences from an unknown apicomplexan group. Node support values correspond to posterior probabilities and consensus support (%) obtained from Bayesian inference and Maximum Likelihood analyses, respectively. Major lineages and respective hosts are highlighted. The newly generated sequences are indicated in bold. Only unique haplotypes were included in the analysis. Host species and infected organs or tissues were detailed for aquatic hosts

The *Goussia* spp. sequence alignment was 510 bp and was analysed using the HKY + I + G model of evolution. The resultant phylogram is depicted in Fig. 1 and included sequences from the heart and kidney of *S. japonicus*, the intestine of farmed *D. labrax* and *Sparus aurata*, and farm-associated *Diplodus sargus*. The *Eimeria* spp. and *Calyptospora* spp. alignment included 627 bp and was analysed using the HKY + I + G model of evolution. This phylogram showed two sequences retrieved from *S. canicula* as basal in relation to sequences for other Eimeriidae (Fig. 2). Sequences from parasite lineages found in several organs of *T. luscus*, *T. trachurus* and *P. caeruleostictus* formed a well-supported clade also containing *Calyptospora* species, with the latter two being recovered as sister lineages with high bootstrap support (99% posterior probability and 100 bootstrap support). Additionally, the sequence from a parasite found in the intestine of *T. luscus* as well as sequences obtained from parasites in intestines of farmed *D. labrax*, were closely related to *Eimeria* species found in the gut of other fish species (Fig. 2). The fourth group alignment, herein designated as *Caryospora*-like for the sake of simplicity, contained 512 bp and was analysed using the GTR + I + G model of evolution. The resulting phylogram showed the sequences from *S. canicula* forming a well-supported clade which was basal in the group (Fig. 3). Finally, for the unnamed Apicomplexa, the alignment included 524 bp and used the HKY + G model of evolution. This phylogram showed that some parasite genotypes found in *P. caeruleostictus* and *S. senegalensis* formed a clade that included parasite found in a reef fish and also in the water column of the Caribbean Sea (Fig. 4). This clade appeared as the sister group to several parasite lineages found in corals from the same region (Fig. 4).

The uncorrected p-distances calculated for sequence pairs in each group are presented in supplementary material (Additional file 3: Tables S1-S5). This genetic distance was chosen to allow comparisons across all taxonomic groups. Based on these results, and on the minimum and maximum divergence found between two accepted species in each group, we determined if each new sequence likely corresponded to a species or simply to a lineage within a species. For example, the divergence between accepted species of *Goussia* varied between 0.4–5.8%, so the lineages found in *S. japonicus* and *D. labrax* likely belong to three different species (minimum p-distance = 0.4%, see Additional file 3: Table S1). On the other hand, sequences of *Goussia* spp. recovered from the intestines of *D. sargus* and *S. aurata* were identical and likely corresponded to a single species different from the species for which data are currently available (p-distance between this lineage and other sequenced species varied between 2.2–5.8%). Divergence between the three previously sequenced *Calyptospora* species ranged between 5.2–7.2% (Additional file 3: Table S2), which led us to consider that the lineages from *T. trachurus* may belong to a single species (p-distance ranged between 0.2–1.2%), as well as the lineages found in *P. caeruleostictus* (p-distance ranged between 0.2–0.4%) and *T. luscus* (p-distance ranged between 0 and 1.9%). In the *Eimeria* group, the p-distance found between accepted *Eimeria* species included in our analysis ranged between 4.0–11.2%. However, *Eimeria* species described from birds, for which more data are available, can diverge by less than 1%, so these sequences could be from other species (e.g. *E. tenella* and *E. necatrix* deposited on GenBank under accession numbers KT184354 and DQ136185). Accordingly, although two of the lineages found in *D. labrax* were related with *E. percae* (p-distance varied between 1.3–1.4%), it is likely they belong to a different species, whereas a third lineage recovered from wild and farmed *D. labrax* likely corresponds to *E. variabilis* (p-distance of 0.6%). The apicomplexans sequenced from *S. canicula* were basal in the phylogeny so we cannot ascertain if they belong to the genus *Eimeria* or a close relative; however, they correspond to two different species (p-distance between them of 8.5%), also quite distinct from the taxa included in our analysis (minimum distance of 8.8%). In the unknown clade, it is more difficult to establish a criterion to determine species as taxa remain unclassified. However, taking into account the previous criteria the lineages in *P. caeruleostictus* may correspond to the same species (p-distance of 0.2%), as do the lineages found in *S. senegalensis* (p-distance ranged between 0–0.8%).

## Discussion

The estimates of 18S rRNA phylogenetic relationships proposed in this study are similar to those previously published for these apicomplexan fish pathogens. Briefly, the epicellular *Goussia* (Fig. 1) forms a distinct group, positioned as basal in relation to other *Goussia* groups [7], and members of *Caryospora*, *Isospora* (both Eimeriidae) and *Schellakia* (Schellakiadae) are closely related [27–29]. Previously, *Eimeria* spp*.* from fish and other hosts were placed within the same clade [6, 7], whereas in the current study fish-infecting *Eimeria* formed a distinct clade (Additional file 1: Figure S1; Additional file 2: Figure S2). In our opinion, this discrepancy is due to differences in the 18S rRNA gene fragments employed and the wider range of taxa in our analysis, including members from an undescribed Apicomplexa group.

The criteria used here to consider as putative species the lineages with less divergence (based on p-distance) than the minimum calculated between accepted species, implies that *Eimeria* cf. *variabilis* was encountered in *D. labrax* (see Fig. 2). Although sequence data are becoming essential tools for studying the Apicomplexa (e.g. [7, 30–33]), the strict use of molecular data for species identification can be hampered in the absence of morphological characterisation and requires this results should be interpreted with caution (e.g. [34]). Additionally, the 18S rRNA gene was shown to have limited resolution in *Eimeria* species discrimination in comparison with the mitochondrial cytochrome *c* oxidase subunit 1 gene [30]. In this respect, ours is a conservative approach based on marker choice.

Our data also showed that potential intraspecific variation can be high. For example, divergence between the genotypes in *T. trachurus* reached 1.2% and those infecting *T. luscus* reached 1.9%. This is in line with previous results [8] where intraspecific variation could be as high as 4.2% in *Calyptospora spinosa*. Divergent 18S rRNA paralogs have also been reported to occur within other coccidian species (e.g. 1.3–1.7% for *Eimeria mitis* [35]; *Plasmodium* spp. [36]) and even within individuals (*Eimeria meleagrimitis*, 2.3% [37]). However, many studies have shown that the existence of paralogs are not common, but rather the exception [38, 39].

Regarding the differentiation of wild *vs* fish-farm infecting lineages, only two of the four parasites infecting famed fish, i.e. one *Eimeria* sp. and one *Goussia* sp. (Figs. 1 and 2), were exclusively found in fish farms. A third parasite lineage, likely *Eimeria* cf. *variabilis*, was recovered in farmed and reported from wild *D. labrax*; and a *Goussia* sp. infected both farmed *S. aurata* and farm-associated *D. sargus*. Although this is speculative as both *S. aurata* and *D. sargus* can be natural reservoirs for this parasite, spillover may be occurring from wild to farmed hosts since this parasite was found in only one individual of *S. aurata*, out of 16 analysed hosts, and in two farm-associated *D. sargus*, out of 5 individuals analysed (2 out of 5).

### Epicellular *Goussia* parasitic lineages infecting fish from the North-East Atlantic

Four genotypes closely related with epicellular *Goussia* species *sensu* Rosenthal et al. [7] were recovered among the analised fish. According to the genetic distances within this *Goussia* group (where interspecific distances range between 0.4–4.4%), the genotypes found in *Scomber japonicus*, *Dicentrarchus labrax* and *Sparus aurata* + *Diplodus sargus* could correspond to different species as genetic distances between them ranged between 0.4–7.3%. To the best of our knowledge, besides *G. clupearum* there are no records of other coccidia infecting *Diplodus* spp. On the other hand, both *Goussia sparis* and *Eimeria spari* have been described from the intestine of *S. aurata*; hence, the genotypes retrieved could correspond to any of these parasites.

The species found in *S. japonicus* were closely related to another coccidian species described from the spleen and kidneys of *Lutjanus kasmira*, *Selar crumenophthalmus* and *Mulloidichthys* sp. in Hawaii (only 0.4% divergence, see [8] and [11]), which Whipps et al. [8] suggested to belong to *Goussia*. The morphology of this putative *Goussia* sp. is very similar to that of the coccidian infecting the kidney of *S. aurata* from the Red Sea (originally reported as an haemogregarine [40]) and a *Liza* host species from Africa (also reported as an haemogregarine [41]), suggesting that this putative *Goussia* *sensu* Whipps et al. [8], might have a wide host and geographical range. Although the *Goussia* found in *S. japonicus* could belong to a distinct *Goussia* species, the levels of divergence between the two are at the lower interval of the observed interspecific divergence for this group.

### *Eimeria*, *Calyptospora* and other related parasites infecting fish from the North-East Atlantic

Phylogenetically, *Eimeria* and *Calyptospora* are closely related genera often recovered as sister clades [6, 8]. However, their phenotypes and life-traits are quite distinct (e.g. [42]). While *Eimeria* spp. are mostly described to infect the gut of fish, although with a few exceptions [6, 7], *Calyptospora* infects the liver of their hosts (e.g. [8]). Here, we found three genotypes that likely correspond to three *Eimeria* spp., in the intestine of two in *D. labrax* and one in *T. luscus*. One of the *Eimeria* sp. infecting *D. labrax* is likely to correspond to *Eimeria variabilis* as it was only 0.6% divergent from one sequenced from a *Taurulus bubalis* sampled off the United Kingdom. The other lineages of *Eimeria* were obtained from *D. labrax*, and are closely related to *Eimeria percae* (minimum 0.13% divergence) from the freshwater host *Perca fluviatilis* captured from freshwater habitats in Hungary [7]. So far, only two *Eimeria* species are reported from the intestine of *D. labrax*: *E. dicentrarchi* from wild and farmed fish (e.g. [43, 44]) and *E. bouixi* [44]. However, there are also reports of an unidentified *Eimeria* species in the intestine of wild *D. labrax* off the coasts of Portugal [45]. As such, the lineages here detected could also correspond to these species.

The *Eimeria* sp. sequenced from *T. luscus* is related to *Eimeria nemethi* (4.4% divergence) detected in the freshwater host *Alburnus alburnus* captured in Hungary [7]. Additionally, we found other divergent parasite genotypes infecting the heart, stomach, spleen and intestine of *T. luscus*, which based on our data are more closely related to *Calyptospora* and *Goussia clupearum*, originally described as *Eimeria clupeaurum,* sequenced from *Clupea pallasii*. Similarly, in *T. trachurus* several parasitic genotypes, recovered as closely related with *Calyptospora* and *G. clupearum*, were found in host liver, stomach and intestine. The phylogenetic proximity of *G. clupearum* with *Calyptospora* has already been reported by Friend et al. [12] and confirms the high taxonomic uncertainty associated with members of *Goussia*. So far, *Goussia lusca* is the only coccidian parasite reported to infect *T. luscus*, and this parasite shares morphological similarities in some developmental stages with *G. clupearum* (see [46]). However, the liver seems to be the only tissue affected by *G. lusca*. In turn, *G. clupearum* has been described from several hosts including *T. trachurus* captured off the Galician coast (Spain) [47], and other *Trisopterus* species (e.g. off Scotland [48]). Despite the considerable morphological variation found in *G. clupearum* from different hosts (see [46]), which could indicate the existence of a species complex, infections by *G. clupearum* seem to be restricted to host liver. In *T. trachurus*, besides *G. clupearum*, another extraintestinal parasite, *Goussia cruciata* (originally described as *Eimeria cruciata*) has been reported at high prevalence from this fish species in the Alboran Sea [49] and North-East Atlantic [47]. *Goussia cruciata* has a broader tissue tropism, infecting multiple organs (liver, intestine and pancreas, e.g. [50, 51]). As such it is possible that the parasite lineages retrieved from *T. trachurus* in the present work correspond to this species. This uncertainty might be resolved with morphological characterization of these parasites.

The genotypes sequenced from *Pagrus caeruleostictus* were recovered in the present phylogenetic analysis as sister to the genus *Calyptospora.* To the best of our knowledge, the only coccidians known to infect this fish belong to *Eimeria* (e.g. [52])*.* Although the divergence between the genotypes found in *P. caeruleostictus* and *Calysptospora* spp. ranges between 0.9–11.0%, i.e. above the observed divergence between *Calyptospora* spp. (5.7–7.2%), the monophyly of the clade was highly supported (99% posterior probability and 100 bootstrap support); for this reason we considered the genotypes recovered from *P. caeruleostictus* likely to belong to *Calyptospora*.

Some of the parasites found infecting the liver of *S. canicula* were also related to *Eimeria*. So far *Eimeria lucida* is the only coccidian described to infect *Scyliorhinus canicula*, as well as other elasmobranchs. Hence the *Eimeria* sp. and the Eimeriidae gen. sp. sequences recovered for this host herein, may correspond to *E. lucida.*

### The Apicomplexa infecting *Scyliorhinus canicula* are closely related with the Eimeriidae and Schellakiidae infecting reptiles, amphibians and birds

Some of the Apicomplexa sequenced from *S. canicula* were recovered in the present phylogenetic analysis as sister to coccidia from the Eimeriidae (*Isospora*, *Caryospora*) and Schellakiidae (*Schellakia*) that infect amphibians and reptiles, including marine turtles (Fig. 3). Given the phylogenetic affinities of the available sequences attributed to *Caryospora cheloniae*, (herein designated as *Caryospora* cf. *cheloniae*), it has been suggested that the taxonomy of this parasite should be redefined and that it should be placed in a different genus [29]. Likewise, the genetic divergence between the sequences recovered from *S. canicula* and their relatives also suggests that these should be part of a new genus.

Interestingly, the results we obtained for the phylogeny of these parasite groups match the estimates of host phylogenetic relationships, as elasmobranchs are one of the oldest vertebrates sharing a common ancestor with amphibians, reptiles plus birds and mammals (e.g. [53]). However to confirm this hypothesis more molecular data regarding Apicomplexa infecting fishes and elasmobranchs is needed.

### Undescribed diversity of the Apicomplexa in the marine realm

The work of Janouškovec et al. [4] highlighted the understudied nature of marine and freshwater environments in terms of the Apicomplexa and other related parasites. In the present study, the parasites found in *Solea senegalensis* and *Pagrus caeruleostictus* were close relatives of an apicomplexan from the blood of a Caribbean fish (*Ophioblennius macclurei*, see [13]) and also of a parasite sequenced from an eDNA seawater sample from the Caribbean [54]. These sequences were in turn sister to an unnamed clade composed of parasites recovered from the mucous of several Caribbean corals (see the supplementary material in [4]). The phylogeny of Janouškovec et al. [4] identified these coral-dwelling lineages as sister to *Sarcocystis* and eimeriids, and the authors suggested that they remained undescribed because coral reefs are poorly surveyed for the Apicomplexa. Here we demonstrate that relatives of the lineages found by Janouškovec et al. [4] parasitise common and commercially valuable fish, thus confirming the understudied nature of this parasite group in the marine realm. Morphological data will help clarify the taxonomy of these parasites, but this requires fresh tissue which was not compatible with our sampling strategy.

## Conclusions

Here we demonstrate that the Apicomplexa infecting marine fish, which could be separated into five distinct groups according to their phylogenetic affinities. Three of these, *Goussia*, *Eimeria* and *Calyptospora*, are common coccidian parasites of fish. Another group was related to the genera *Caryospora* (Eimeriidae), *Schellakia* (Schellakiidae) and *Isospora* (Eimeriidae), which typically infect terrestrial vertebrates (e.g. [55]). Lastly, a set of sequences were placed in an unknown group of the Apicomplexa so far only recovered from the Caribbean Sea, having been found in the mucus of corals [4], an environmental sample from the water column [54], and a fish [13]. Additionally, our phylogenetic results suggest that the Apicomplexa recovered from elasmobranchs are basal, which is in line with the host’s phylogenetic relationships since elasmobranchs (together with the holocephalans) are the oldest jawed vertebrates on earth (e.g. [56]).

## Additional files

Additional file 1: Figure S1. Bayesian inference analysis of epicellular *Goussia* spp. and the unknown apicomplexan clade based on sequences generated in the present study plus previously public sequences retrieved from GenBank. The newly generated sequences are depicted in bold. Host species and infected organs or tissues were detailed for all aquatic hosts. (EPS 3990 kb)

Additional file 2: Figure S2. Bayesian inference analysis of dispersed and nodular *Goussia*, *Caryospora*-, *Calyptospora*- and *Eimeria*-like sequences generated in the present study, plus previously published or public sequences retrieved from GenBank. Node support values correspond to posterior probabilities and consensus support (%) obtained from Bayesian inference and Maximum Likelihood analyses, respectively. The major phylogenetic groups infecting aquatic hosts are highlighted according to the legend, and the newly generated sequences are indicated in bold. Host species and infected organs or tissues were detailed for all aquatic hosts. (EPS 3133 kb)

Additional file 3: Table S1. Uncorrected p-distances estimated between pairs of samples fror the *Goussia* group. Sequences generated in the present study are highlighted in bold, as well as p-distances between them. Organs from where the sequences were retrieved were coded as follows: * intestine, ˥ gall bladder, Г liver,†stomach, ‡spleen*, •* heart, α kidney. **Table S2.** Uncorrected p-distances between pairs of sequences from the *Calyptospora* group. Sequences generated in this study are highlighted in bold**,** as well as p-distances between them. Organs from where the sequences were retrieved were coded as follows: * intestine, ˥ gall bladder, Г liver, ‡spleen*, •* heart, α kidney. **Table S3.** Uncorrected p-distances between pairs of sequences from the *Eimeria* group. Sequences generated in this study are highlighted in bold**,** as well as p-distances between them. All sequences were obtained from the intestines (coded as *) or liver (coded as Г). **Table S4.** Uncorrected p-distances between pairs of sequences in the *Caryospora*, *Shellackia* and *Isospor*a group. Sequences generated in this study are highlighted in bold**,** as well as p-distances between them. Organs from where the sequences were retrieved were coded as follows: *anal gland, Г liver,†stomach. **Table S5.** Uncorrected p-distances between pairs of sequences in the Unknown Apicomplexa group. Sequences generated in this study are highlighted in bold**,** as well as p-distances between them. Organs from where the sequences were retrieved were coded as follows: * intestine, ˥ gall bladder, Г liver,†stomach, ‡spleen*, •* heart, α kidney, ф testicle, ¥ gill. (DOCX 38 kb)
