# Supplementary material for: Phylogenetic analysis of apicomplexan parasites infecting commercially valuable species from the North-East Atlantic reveals high levels of diversity and insights into the evolution of the group
Source: Parasit Vectors. 2018 Jan 25;11:63. doi: 10.1186/s13071-018-2645-7 (PMC5785827; doi:10.1186/s13071-018-2645-7)
Supplement: Supplementary file 3 — Uncorrected p-distances estimated between pairs of samples fror the Goussia group. Sequences generated in the present study are highlighted in bold, as well as p-distances between them. Organs from where the sequences were retrieved were coded as follows: * intestine, ˥ gall bladder, Г liver,†stomach, ‡spleen, • heart, α kidney. Table S2. Uncorrected p-distances between pairs of sequences from the Calyptospora group. Sequences generated in this study are highlighted in bold, as well as p-distances between them. Organs from where the sequences were retrieved were coded as follows: * intestine, ˥ gall bladder, Г liver, ‡spleen, • heart, α kidney. Table S3. Uncorrected p-distances between pairs of sequences from the Eimeria group. Sequences generated in this study are highlighted in bold, as well as p-distances between them. All sequences were obtained from the intestines (coded as *) or liver (coded as Г). Table S4. Uncorrected p-distances between pairs of sequences in the Caryospora, Shellackia and Isospora group. Sequences generated in this study are highlighted in bold, as well as p-distances between them. Organs from where the sequences were retrieved were coded as follows: *anal gland, Г liver,†stomach. Table S5. Uncorrected p-distances between pairs of sequences in the Unknown Apicomplexa group. Sequences generated in this study are highlighted in bold, as well as p-distances between them. Organs from where the sequences were retrieved were coded as follows: * intestine, ˥ gall bladder, Г liver,†stomach, ‡spleen, • heart, α kidney, ф testicle, ¥ gill. (DOCX 38 kb) [file 13071_2018_2645_MOESM3_ESM.docx]

|  | ***Goussia sp*** *•*  (*S. japonicus*) | ***Goussia sp*** *α*  (*S. japonicus*) | *Goussia*  sp HM117907 | ***Goussia sp* ***  **(***D. sargus)* | ***Goussia sp****  **(***D. sargus)* | ***Goussia sp****  **(***S. aurata)* | ***Goussia sp****  **(***D. labrax)* | *G. szekelyi*  GU479656 | *G. pannonica*  GU479651 | *G. koertingi*  GU479647 | *G. janae*  AY043206 | *G.ameliae*  KP411007 |
| --- | --- | --- | --- | --- | --- | --- | --- | --- | --- | --- | --- | --- |
| ***Goussia sp*** *α*  (*S.japonicus*) | **0,004** |  |  |  |  |  |  |  |  |  |  |  |
| *Goussia*  sp HM117907 | 0,004 | 0,008 |  |  |  |  |  |  |  |  |  |  |
| ***Goussia sp* ***  **(***D. sargus)* | **0,054** | **0,053** | 0,050 |  |  |  |  |  |  |  |  |  |
| ***Goussia sp* ***  **(***D. sargus)* | **0,054** | **0,053** | 0,050 | **0,000** |  |  |  |  |  |  |  |  |
| ***Goussia sp****  **(***S. aurata)* | **0,054** | **0,053** | 0,050 | **0,000** | **0,000** |  |  |  |  |  |  |  |
| ***Goussia sp****  **(***D. labrax)* | **0,050** | **0,049** | 0,047 | **0,073** | **0,073** | **0,073** |  |  |  |  |  |  |
| *G. szekelyi*  GU479656 | 0,052 | 0,051 | 0,048 | 0,028 | 0,028 | 0,026 | 0,073 |  |  |  |  |  |
| *G. pannonica*  GU479651 | 0,047 | 0,047 | 0,044 | 0,022 | 0,022 | 0,020 | 0,069 | 0,016 |  |  |  |  |
| *G. koertingi*  GU479647 | 0,047 | 0,047 | 0,044 | 0,026 | 0,026 | 0,024 | 0,069 | 0,020 | 0,004 |  |  |  |
| *G. janae*  AY043206 | 0,047 | 0,047 | 0,044 | 0,028 | 0,028 | 0,026 | 0,071 | 0,022 | 0,004 | 0,006 |  |  |
| *G.ameliae*  KP411007 | 0,039 | 0,037 | 0,040 | 0,038 | 0,038 | 0,036 | 0,067 | 0,040 | 0,036 | 0,036 | 0,036 |  |
| *E. leucisci*  GU479649 | 0,043 | 0,047 | 0,042 | 0,048 | 0,048 | 0,048 | 0,077 | 0,044 | 0,040 | 0,044 | 0,044 | 0,058 |

**Additional file 3: Table S1** Uncorrected p-distances estimated between pairs of samples fror the *Goussia* group. Sequences produced in the present study are highlighted in bold, as well as p-distances between them. Organs from where the sequences were retrieved were coded as follows: * intestine, ˥ gall bladder, Г liver,†stomach, ‡spleen*, •* heart, α kidney

**Table S2** Uncorrected p-distances between pairs of sequences from the *Calyptospora* group. Sequences produced in this study are highlighted in bold**,** as well as p-distances between them. Organs from where the sequences were retrieved were coded as follows: * intestine, ˥ gall bladder, Г liver, ‡spleen*, •* heart, α kidney

|  | **Eimeriidae gen. sp**. *****  **(*T. trachurus*)** | ***Calyptospora***  **sp ˥**  **(*P.caeruleostictus*)** | ***Calyptospora***  **sp***  **(*P.caeruleostictus*)** | ***Calyptospora***  **spГ**  **(***P.caeruleostictus***)** | *C. serrasalmi*  FJ904638 | *C. funduli* FJ904646 | *C. spinosa* FJ904635 | *G. clupearum*  KT025256*)* | **Eimeriidae**  **gen. sp†**  **(*T.luscus*)** | **Eimeriidae gen. sp˥**  ***(T.luscus)*** | **Eimeriidae gen. sp ***  ***(T.luscus)*** | **Eimeriidae gen. sp ***  ***(T.luscus)*** | **Eimeriidae gen. sp Г**  ***(T.luscus)*** | **Eimeriidae gen. sp ˥**  ***(T.luscus)*** | **Eimeriidae gen. sp Г**  ***(T.luscus)*** | **Eimeriidae gen. sp***  ***(T.luscus)*** | **Eimeriidae gen. sp ‡ *(T.luscus)*** | **Eimeriidae gen. sp •**  ***(T.luscus)*** | **Eimeriidae**  **gen. sp Г**  **(*T. trachurus*)** | **Eimeriidae gen. sp ***  **(*T. trachurus*)** | **Eimeriidae gen. sp †**  **(*T. trachurus*)** | **Eimeriidae sp†**  **(*T. trachurus)*** |
| --- | --- | --- | --- | --- | --- | --- | --- | --- | --- | --- | --- | --- | --- | --- | --- | --- | --- | --- | --- | --- | --- | --- |
| ***Calyptospora***  **sp˥**  ***(P.caeruleostictus)*** | **0,164** |  |  |  |  |  |  |  |  |  |  |  |  |  |  |  |  |  |  |  |  |  |
| ***Calyptospora***  **sp*****  ***(P.caeruleostictus)*** | **0,164** | **0,004** |  |  |  |  |  |  |  |  |  |  |  |  |  |  |  |  |  |  |  |  |
| ***Calyptospora***  **sp*Г***  ***(P.caeruleostictus)*** | **0,164** | **0,002** | **0,002** |  |  |  |  |  |  |  |  |  |  |  |  |  |  |  |  |  |  |  |
| *C. serrasalmi* FJ904638 | 0,171 | 0,090 | 0,093 | 0,093 |  |  |  |  |  |  |  |  |  |  |  |  |  |  |  |  |  |  |
| *C. funduli* FJ904646 | 0,187 | 0,106 | 0,109 | 0,110 | 0,053 |  |  |  |  |  |  |  |  |  |  |  |  |  |  |  |  |  |
| *C. spinosa* FJ904635 | 0,176 | 0,099 | 0,103 | 0,103 | 0,052 | 0,072 |  |  |  |  |  |  |  |  |  |  |  |  |  |  |  |  |
| G. clupearum KT025256 | 0,157 | 0,128 | 0,132 | 0,134 | 0,120 | 0,137 | 0,136 |  |  |  |  |  |  |  |  |  |  |  |  |  |  |  |
| **Eimeriidae gen. sp** †  (*T.luscus*) | **0,191** | **0,152** | **0,154** | **0,152** | 0,153 | 0,170 | 0,161 | 0,171 |  |  |  |  |  |  |  |  |  |  |  |  |  |  |
| ***Coccidia*** sp˥  *(T.luscus)* | **0,172** | **0,139** | **0,141** | **0,139** | 0,138 | 0,153 | 0,147 | 0,156 | **0,002** |  |  |  |  |  |  |  |  |  |  |  |  |  |
| **Eimeriidae gen. sp** *  *(T.luscus)* | **0,187** | **0,149** | **0,152** | **0,149** | 0,151 | 0,170 | 0,158 | 0,168 | **0,006** | **0,004** |  |  |  |  |  |  |  |  |  |  |  |  |
| **Eimeriidae gen. sp** sp*  *(T.luscus)* | **0,172** | **0,137** | **0,139** | **0,137** | 0,137 | 0,156 | 0,145 | 0,154 | **0,004** | **0,002** | **0,004** |  |  |  |  |  |  |  |  |  |  |  |
| **Eimeriidae gen. sp** Г  *(T.luscus)* | **0,170** | **0,138** | **0,140** | **0,138** | 0,139 | 0,157 | 0,148 | 0,157 | **0,002** | **0,000** | **0,002** | **0,000** |  |  |  |  |  |  |  |  |  |  |
| **Eimeriidae gen. sp** ˥  *(T.luscus)* | **0,183** | **0,146** | **0,148** | **0,146** | 0,147 | 0,166 | 0,155 | 0,164 | **0,004** | **0,002** | **0,002** | **0,002** | **0,000** |  |  |  |  |  |  |  |  |  |
| **Eimeriidae gen. sp** Г  *(T.luscus)* | **0,170** | **0,133** | **0,135** | **0,134** | 0,132 | 0,151 | 0,141 | 0,149 | **0,002** | **0,000** | **0,002** | **0,000** | **0,000** | **0,000** |  |  |  |  |  |  |  |  |
| **Eimeriidae gen. sp** *  *(T.luscus)* | **0,170** | **0,132** | **0,136** | **0,137** | 0,131 | 0,150 | 0,140 | 0,148 | **0,002** | **0,002** | **0,000** | **0,002** | **0,000** | **0,000** | **0,002** |  |  |  |  |  |  |  |
| **Eimeriidae gen. sp** ‡ *(T.luscus)* | **0,177** | **0,149** | **0,151** | **0,149** | 0,150 | 0,167 | 0,159 | 0,168 | **0,019** | **0,015** | **0,019** | **0,013** | **0,013** | **0,017** | **0,013** | **0,013** |  |  |  |  |  |  |
| **Eimeriidae gen. sp** •  *(T.luscus)* | **0,203** | **0,205** | **0,208** | **0,205** | 0,207 | 0,227 | 0,218 | 0,222 | **0,018** | **0,013** | **0,018** | **0,011** | **0,011** | **0,015** | **0,011** | **0,011** | **0,008** |  |  |  |  |  |
| **Eimeriidae gen. sp** Г  (*T. trachurus*) | **0,012** | **0,130** | **0,130** | **0,130** | 0,131 | 0,148 | 0,135 | 0,129 | **0,150** | **0,135** | **0,147** | **0,134** | **0,136** | **0,144** | **0,133** | **0,135** | **0,147** | **0,203** |  |  |  |  |
| **Eimeriidae gen. sp** *  (*T. trachurus*) | **0,012** | **0,130** | **0,130** | **0,131** | 0,131 | 0,148 | 0,136 | 0,125 | **0,150** | **0,135** | **0,147** | **0,134** | **0,136** | **0,144** | **0,130** | **0,132** | **0,147** | **0,203** | **0,004** |  |  |  |
| **Eimeriidae gen. sp** †  (*T. trachurus*) | **0,007** | **0,137** | **0,137** | **0,137** | 0,140 | 0,154 | 0,145 | 0,134 | **0,152** | **0,139** | **0,150** | **0,139** | **0,138** | **0,146** | **0,138** | **0,140** | **0,149** | **0,201** | **0,006** | **0,006** |  |  |
| **Eimeriidae gen. sp** †  (*T. trachurus)* | **0,010** | **0,129** | **0,129** | **0,129** | 0,131 | 0,148 | 0,135 | 0,124 | **0,147** | **0,133** | **0,145** | **0,132** | **0,134** | **0,142** | **0,129** | **0,132** | **0,145** | **0,201** | **0,002** | **0,002** | **0,004** |  |
| **Eimeriidae gen. sp** Г  (*T. trachurus*) | **0,010** | **0,124** | **0,124** | **0,127** | 0,129 | 0,146 | 0,133 | 0,123 | **0,147** | **0,135** | **0,145** | **0,134** | **0,134** | **0,142** | **0,130** | **0,130** | **0,145** | **0,201** | **0,004** | **0,004** | **0,004** | **0,002** |

**Table S3** Uncorrected p-distances between pairs of sequences from the *Eimeria* group. Sequences produced in this study are highlighted in bold**,** as well as p-distances between them. All sequences were obtained from the intestines (coded as *) or liver (coded as Г)

|  | ***Eimeria*** sp Г  (S. canicula) | ***Eimeria*** sp*  (*T.luscus*) | *E. nemethi*  (GU479634) | *E. variabilis*  (GU479674) | ***Eimeria*** sp*  (*D. labrax*) | ***Eimeria*** sp*  (*D. labrax*) | ***Eimeria*** sp*  (*D. labrax*) | *E. percae*  *(*GU479663) | *E.anguillae*  (GU479633) |
| --- | --- | --- | --- | --- | --- | --- | --- | --- | --- |
|  |  |  |  |  |  |  |  |  |  |
| ***Eimeria*** sp*  (*T.luscus*) | **0,102** |  |  |  |  |  |  |  |  |
| *E. nemethi*  (GU479634) | 0,093 | 0,055 |  |  |  |  |  |  |  |
| *E. variabilis*  (GU479674) | 0,093 | 0,120 | 0,108 |  |  |  |  |  |  |
| **Eimeria** sp*  (*D. labrax*) | **0,093** | **0,118** | 0,108 | 0,006 |  |  |  |  |  |
| **Eimeria** sp*  (*D. labrax*) | **0,088** | **0,120** | 0,108 | 0,043 | **0,045** |  |  |  |  |
| **Eimeria** sp*  (*D. labrax*) | **0,088** | **0,120** | 0,116 | 0,046 | **0,048** | **0,002** |  |  |  |
| *E. percae*  *(*GU479663) | 0,093 | 0,129 | 0,112 | 0,040 | 0,043 | 0,014 | 0,013 |  |  |
| *E.anguillae*  (GU479633) | 0,115 | 0,129 | 0,110 | 0,053 | 0,055 | 0,047 | 0,048 | 0,047 |  |
| ***Eimeria*** sp Г  (S. canicula) | **0,085** | **0,129** | 0,112 | 0,124 | **0,122** | **0,118** | **0,125** | 0,122 | 0,141 |

|  |  |  |  |  |  |  |  |  |  |
| --- | --- | --- | --- | --- | --- | --- | --- | --- | --- |

|  | *C. bigenetica* AF06097 | ***Coccidia sp*** Г ***(S. canicula)*** | ***Coccidia sp* † *(S. canicula*** | ***Coccidia sp*******(S. canicula*)** | ***Coccidia sp*******(S. canicula* †** | *I.*  *manorinae* KT224379 | *Isospora* sp KF648871 | *E.ranae* EU7172191 | *S.bolivari* KJ131415 | *Schellackia* sp. JX984674 | *Schellackia* sp KJ189384 | *E. arnyi* AY613853 | *I. gekkonis* KU180246 | Eimeriidae sp KT956976 | Eimeriidae sp KT956977 | *C. cheloniae* KT361639 | *C. cheloniae*  KT361640 |
| --- | --- | --- | --- | --- | --- | --- | --- | --- | --- | --- | --- | --- | --- | --- | --- | --- | --- |
| ***Coccidia sp***  ***S. canicula*** Г | 0.085 |  |  |  |  |  |  |  |  |  |  |  |  |  |  |  |  |
| ***Coccidia sp***  ***S. canicula* †** | 0.089 | **0.000** |  |  |  |  |  |  |  |  |  |  |  |  |  |  |  |
| ***Coccidia sp***  ***S. canicula*** * | 0.077 | **0.008** | **0.009** |  |  |  |  |  |  |  |  |  |  |  |  |  |  |
| ***Coccidia sp***  ***S. canicula*** * | 0.079 | **0.010** | **0.011** | **0.002** |  |  |  |  |  |  |  |  |  |  |  |  |  |
| *I. manorinae* KT224379 | 0.048 | 0.074 | 0.076 | 0.066 | 0.068 |  |  |  |  |  |  |  |  |  |  |  |  |
| *Isospora* sp KF648871 | 0.040 | 0.074 | 0.076 | 0.068 | 0.070 | 0.014 |  |  |  |  |  |  |  |  |  |  |  |
| *E. ranae* EU7172191 | 0.076 | 0.078 | 0.085 | 0.076 | 0.078 | 0.074 | 0.070 |  |  |  |  |  |  |  |  |  |  |
| *S. bolivari* KJ131415 | 0.070 | 0.062 | 0.068 | 0.058 | 0.060 | 0.062 | 0.058 | 0.042 |  |  |  |  |  |  |  |  |  |
| *Schellackia* sp. JX984674 | 0.068 | 0.062 | 0.068 | 0.056 | 0.058 | 0.060 | 0.056 | 0.046 | 0.018 |  |  |  |  |  |  |  |  |
| *Schellackia* sp KJ189384 | 0.066 | 0.064 | 0.070 | 0.058 | 0.060 | 0.058 | 0.054 | 0.048 | 0.020 | 0.008 |  |  |  |  |  |  |  |
| *E. arnyi* AY613853 | 0.062 | 0.054 | 0.059 | 0.048 | 0.050 | 0.056 | 0.048 | 0.042 | 0.018 | 0.016 | 0.020 |  |  |  |  |  |  |
| *I. gekkonis* KU180246 | 0.044 | 0.094 | 0.096 | 0.086 | 0.088 | 0.050 | 0.048 | 0.088 | 0.084 | 0.082 | 0.080 | 0.078 |  |  |  |  |  |
| Eimeriidae sp KT956976 | 0.076 | 0.083 | 0.091 | 0.082 | 0.083 | 0.074 | 0.078 | 0.078 | 0.060 | 0.060 | 0.064 | 0.062 | 0.094 |  |  |  |  |
| Eimeriidae.sp. KT956977 | 0.074 | 0.083 | 0.091 | 0.082 | 0.083 | 0.074 | 0.074 | 0.076 | 0.062 | 0.064 | 0.066 | 0.062 | 0.090 | 0.016 |  |  |  |
| *C. cheloniae* KT361639 | 0.071 | 0.057 | 0.063 | 0.051 | 0.053 | 0.060 | 0.055 | 0.082 | 0.058 | 0.031 | 0.027 | 0.031 | 0.024 | 0.066 | 0.066 |  |  |
| *C. cheloniae* KT361640 | 0.084 | 0.099 | 0.110 | 0.095 | 0.097 | 0.082 | 0.086 | 0.100 | 0.084 | 0.069 | 0.071 | 0.073 | 0.071 | 0.026 | 0.018 | 0.069 |  |
| Coccidia sp AY728896 | 0,147 | 0,127 | 0,139 | 0,125 | 0,127 | 0,129 | 0,135 | 0,152 | 0,134 | 0,130 | 0,126 | 0,130 | 0,116 | 0,137 | 0,131 | 0,135 | 0,148 |

**Table S4** Uncorrected p-distances between pairs of sequences in the *Caryospora*, *Shellackia* and *Isospor*a group. Sequences produced in the current study are highlighted in bold**,** as well as p-distances between them. Organs from where the sequences were retrieved were coded as follows: *anal gland, Г liver,†stomach.

**Table S5** Uncorrected p-distances between pairs of sequences in the Unknown Apicomplexa group. Sequences produced in the current study are highlighted in bold**,** as well as p-distances between them. Organs from where the sequences were retrieved were coded as follows: * intestine, ˥ gall bladder, Г liver,†stomach, ‡spleen*, •* heart, α kidney, ф testicle, ¥ gill

|  | **Apicomplexa fam. gen. sp.*•***  **(*P.caeruleostictus*)** | **Apicomplexa fam. gen. sp α**  **(*P.caeruleostictus*)** | **Apicomplexa fam. gen. sp ф**  **(*S.senegalensis*)** | **Apicomplexa fam. gen. sp Г**  **(*S.senegalensis*)** | **Apicomplexa fam. gen. sp ¥**  **(*S.senegalensis*)** | **Apicomplexa fam. gen. sp Г**  **(*S.senegalensis*)** | Apicomplexa sp KT806396 | Apicomplexa sp KP213205 | Apicomplexa sp KP213194 | Apicomplexa sp KP213210 | Apicomplexa sp AF238264 | Apicomplexa sp KP213193 | Apicomplexa sp  KP213250 |
| --- | --- | --- | --- | --- | --- | --- | --- | --- | --- | --- | --- | --- | --- |
| **Apicomplexa fam. gen. sp α**  **(*P.caeruleostictus*)** | **0,002** |  |  |  |  |  |  |  |  |  |  |  |  |
| **Apicomplexa fam. gen. sp ф**  **(*S. senegalensis*)** | **0,018** | **0,008** |  |  |  |  |  |  |  |  |  |  |  |
| **Apicomplexa fam. gen. sp Г**  **(*S. senegalensis*)** | **0,018** | **0,008** | **0,000** |  |  |  |  |  |  |  |  |  |  |
| **Apicomplexa fam. gen. sp ¥**  **(*S.senegalensis*)** | **0,022** | **0,013** | **0,002** | **0,004** |  |  |  |  |  |  |  |  |  |
| **Apicomplexa fam. Gen. sp Г**  **(*S.senegalensis*)** | **0,022** | **0,013** | **0,006** | **0,008** | **0,002** |  |  |  |  |  |  |  |  |
| Apicomplexa sp  KT806396 | 0,032 | 0,023 | 0,024 | 0,026 | 0,028 | 0,028 |  |  |  |  |  |  |  |
| Apicomplexa sp  KP213205 | 0,121 | 0,117 | 0,112 | 0,114 | 0,114 | 0,114 | 0,119 |  |  |  |  |  |  |
| Apicomplexa sp  KP213194 | 0,119 | 0,114 | 0,109 | 0,112 | 0,112 | 0,112 | 0,116 | 0,002 |  |  |  |  |  |
| Apicomplexa sp  KP213210 | 0,121 | 0,117 | 0,119 | 0,122 | 0,119 | 0,119 | 0,129 | 0,052 | 0,049 |  |  |  |  |
| Apicomplexa sp  AF238264 | 0,104 | 0,098 | 0,091 | 0,093 | 0,093 | 0,097 | 0,103 | 0,059 | 0,057 | 0,049 |  |  |  |
| Apicomplexa sp  KP213193 | 0,135 | 0,131 | 0,126 | 0,128 | 0,126 | 0,126 | 0,135 | 0,052 | 0,049 | 0,044 | 0,047 |  |  |
| Apicomplexa sp  KP213250 | 0,131 | 0,127 | 0,122 | 0,124 | 0,122 | 0,121 | 0,131 | 0,047 | 0,045 | 0,040 | 0,042 | 0,005 |  |
| Apicomplexa sp  KP213250 | 0,089 | 0,075 | 0,072 | 0,075 | 0,078 | 0,084 | 0,087 | 0,221 | 0,217 | 0,217 | 0,135 | 0,228 | 0,219 |
